# 

**Published:** 1904-04-16

**Authors:** 


### NOTICE TO CORRESPONDENTS.

All MSS., Letters, Books for Review, and other matters intended for the Editor should be addressed The Editor, "The Hospital," The Hospital Buildings, 28 & 29 Southampton Street, Strand, W.C.

The Editor cannot undertake to return rejected MS., even when accompanied by stamped directed envelope.

**Notice to Advertisers and Subscribers.**

All Advertisements, Orders for Copies of The Hospital, and Business Communications should be addressed to **THE MANAGER (Not to the Editor),** The Hospital, 28 & 29 Southampton Street, Strand, London, W.C.

The Cost of Subscription, post free, is as follows:—Per week, 3½d.; per quarter, 3s. 9d.; per half-year, 7s. 6d.; per annum, 15s. Foreign and Colonial:—Per annum, 19s. 6d., payable, in all cases, in advance.

**NOTICE.—Vol. XXXV. of "The Hospital" (October 1903 to March 1904), in handsome cloth binding, will shortly be on sale at the office of this Journal, price 10s. 6d. Cases for binding the Numbers contained in this Volume, 2s. Index to Vol. XXXV., 3½d., post free.**

**The Monthly part for April is now ready. Price 1s., by post, 1s. 3d.**

### Scraps and Gleanings.

The late Elizabeth Porter, of Halifax, has bequeathed £26,300 to be distributed among various charities, including £2,000 for the Halifax Infirmary.

The Society of Medical Phonographers will hold its annual shorthand examination in May 1904. Two prizes will be offered, each of the value of £3, one for first-year students, and one for students of more than one year's standing. The competition is open, without entrance fee, to any registered medical student in the United Kingdom. Further details may be obtained from Dr. P. G. Griffith, Villa Monitor, Green Lanes, Hornsey, N.

### The Student of Medicine.

### APPOINTMENTS.

**C. J. Barnes**, L.S.A., District Medical Officer of the Chippenham Union. **H. Reynolds Brown**, M.D., C.M.Edin., Medical Officer for the All Saints District of Maldon, and Public Vaccinator for the Purleigh District of the Maldon Union. **J. P. Conway**, L.R.C.P.&S.Edin., L.F.P.S.Glasg., Medical Officer for No. 3 District of the Greenwich Union. **W. S. Griffith**, M.B., C.M.Edin., Certifying Factory Surgeon for the Milford Haven District, Pembroke. **J. H. Harvey**, M.R.C.S., L.R.C.P., District Medical Officer of the Weymouth Union. **L. W. Hignett**, M.B., C.M.Edin., District Medical Officer of the Uxbridge Union. **James Jeffares**, L.R.C.P.&S.Edin., L.F.P.S.Glasg., Certifying Factory Surgeon for the Kegworth District, Leicestershire. **Edward Linton**, L.R.C.P.&S.Edin., L.F.P.S.Glasg., Government Medical Officer and Vaccinator at Wellington, N.S.W. **Allen Muscio**, M.B.Syd., Government Medical Officer and Vaccinator at Emmaville, N.S.W. **L. S. Norman**, M.B., C.M.Edin., District Medical Officer of the South Shields Union. **C. F. Seville**, M.B.Lond., District Medical Officer of the Hunslet Union. **H. M. Stumbles**, M.B., Ch.B.Edin., Certifying Factory Surgeon for the Amble District, Northumberland. **John Lewis Thomas**, M.R.C.S.Eng., L.S.A., Medical Officer of Health to the Brynmawr Urban District Council. **J. R. Hall Walker**, M.D., C.M.Edin., Medical Officer to the Workhouse of the Risbridge Union at Kedington. **D. J. Williams**, M.R.C.S., L.R.C.P.Lond., Chief Resident Medical Officer at the Kingston Asylum, Jamaica. **R. Le Geyt Worsley**, M.R.C.S., L.R.C.P. Lond., L.S.A., Deputy Medical Officer, H.M. Prison, Manchester. **C. W. F. Young**, M.D.Lond., D.P.H.Camb., Medical Officer for the Middlesex County Council.

### Standard Nursing Manuals.

### A HANDBOOK FOR NURSES.

By J. K. Watson, M.D., M.B., C.M.

Second Edition. Crown 8vo. over 400 pp, profusely illustrated, **5s.** net, **5s. 5d.** post free.

"A concise and comprehensive exposition of as much medical knowledge as a nurse needs. The work cannot but prove useful to those for whom it has been designed."
*Scotsman.*

### ELEMENTARY ANATOMY AND SURGERY FOR NURSES.

By William McAdam Eccles, M.S.Lond., M.B., &c.

Crown 8vo. Illustrated, cloth, **2s. 6d.** post free.

"Valuable to nurses who are beginning the study of anatomy. The instruction is given very clearly and concisely, and the reader is able to glean much information in a very condensed form."—*Nursing Notes.*

### NURSING: ITS THEORY AND PRACTICE.

By Percy G. Lewis, M.D., M.R.C.S., L.S.A.

New and Enlarged Edition.

Crown 8vo. over 400 pp. profusely illustrated, **3s. 6d.** post free.

"Nurses will find the book full of interest and instruction, which cannot fail to assist them in their work."
*British Medical Journal.*

### ELEMENTARY PHYSIOLOGY FOR NURSES.

By C. F. Marshall, M.D., B.Sc., F.R.C.S.

Crown 8vo. Illustrated, cloth, **2s.** post free.

"Nurses will find in it all that they require to know of the subjects treated."—*Daily Chronicle.*

### A PRACTICAL HANDBOOK OF MIDWIFERY.

By Francis W. Haultain, M.D.

New and Revised Edition. Illustrated. Handsomely bound in leather, **6s.** net, **6s. 3d.** post free.

"One of the best of its kind, and well fitted to perform the functions its author claims for it."

*Edinburgh Medical Journal.*

### MIDWIVES' POCKET BOOK.

(With Appendix containing particulars and rules of the Central Midwives Board.)

By Honnor Morten.

Small crown 8vo. cloth, **1s.** post free.

An exceedingly useful handbook for those entering for the L.O.S. certificate as well as for qualified Midwives.

### SURGICAL WARD-WORK AND NURSING.

By Alexander Miles, M.D.Edin., C.M., F.R.C.S.E.

Revised Edition. Demy 8vo. Illustrated, cloth gilt, **3s. 6d.** net, **3s. 10d.** post free.

"An illustrated manual which will furnish much needed guidance to the student and the nurse in the early stages of their apprenticeship."—*The Times.*

### ART OF MASSAGE.

By A. Creighton Hale.

Second Edition. Demy 8vo. Profusely illustrated, cloth gilt, **6s.** post free.

"Mrs. Hale seems to have had considerable experience, not only as a practitioner, but as a teacher of the art, and explains in a series of clearly illustrated articles the many complaints for which massage has been found beneficial, and the special manipulations required in each case."
*Morning Post.*

### OPHTHALMIC NURSING.

By Sydney Stephenson, M.B., F.R.C.S.E.

Second Edition. Crown 8vo. illustrated, with glossary of terms, cloth, **3s. 6d.** net, **3s. 10d.** post free.

"May very advantageously be studied by nurses who are about to enter the Ophthalmic service of a hospital."
*Lancet.*

### ART OF FEEDING THE INVALID.

By a Medical Practitioner and a Lady Professor of Cookery.

New and Popular Edition. Demy 8vo. cloth, **1s. 6d.** post free.

"A new edition of this valuable work is very welcome, and we heartily commend it to all who desire reliable and practical instruction in sick cookery."—*Nursing Notes.*

### NURSING IN DISEASES OF THE THROAT, NOSE AND EAR.

By P. Macleod Yearsley, F.R.C.S.Eng., M.R.C.S., L.R.C.P.Lond.

Demy 8vo. Illustrated, cloth, **2s. 6d.** post free.

"Of practical service to every nurse who wishes to be thoroughly trained."
*Scottish Medical and Surgical Journal.*

### PRACTICAL GUIDE TO SURGICAL BANDAGING AND DRESSINGS.

By Wm. Johnson Smith, F.R.C.S.

Demy 16mo., profusely Illustrated (suitable for apron pocket), cloth, **2s.** post free.

*COMPLETE CATALOGUE POST FREE ON APPLICATION.*

The SCIENTIFIC PRESS, LTD., 28 & 29 Southampton Street, Strand, London, W.C.

